# Impact of Site Disturbances from Harvesting and Logging on Soil Physical Properties and *Pinus kesiya* Tree Growth

**DOI:** 10.1155/2014/323626

**Published:** 2014-07-16

**Authors:** Edward Missanjo, Gift Kamanga-Thole

**Affiliations:** Malawi College of Forestry and Wildlife, Private Bag 6, Dedza, Malawi

## Abstract

A study was conducted to determine the impacts of soil disturbance and compaction on soil physical properties and tree growth and the effectiveness of tillage in maintaining or enhancing site productivity for intensively managed *Pinus kesiya* Royle ex Gordon sites in Dedza, Malawi. The results indicate that about fifty-two percent of the area of compacted plots was affected by the vehicular traffic. Seventy percent of the trees were planted on microsites with some degree of soil disturbance. Soil bulk density at 0–20 cm depth increased from 0.45 to 0.66 Mg m^−3^ in the most compacted portions of traffic lanes. Soil strength in traffic lanes increased at all 60 cm depth but never exceeded 1200 kPa. Volumetric soil water content in compacted traffic lanes was greater than that in noncompacted soil. Total soil porosity decreased 13.8% to 16.1% with compaction, while available water holding capacity increased. The study revealed no detrimental effects on tree height and diameter from soil disturbance or compaction throughout the three growing season. At the ages of two and three, a tree volume index was actually greater for trees planted on traffic lanes than those on nondisturbed soil.

## 1. Introduction

Timber harvesting and logging is one of the vital activities carried out in the forestry sector in Malawi. This is because it creates a room for establishment of new stand in the process of removing the old age. This also reduces the impact which may follow if the stands are left unharvested past their harvesting age [1]. These impacts may range from disease attraction to illegal harvesting. Timber harvesting activities cause some degree of soil disturbance [2] on soil physical, chemical, and biological properties; this also reduces site productivity [3].

Ground-based logging systems can cause serious disturbance to the physical properties of forest soil due to soil compaction [4]. Compaction is one of the major causes of soil degradation from logging equipment [5]. Soil compaction decreases porosity and infiltration capacity and increases bulk density and soil strength [4]. Bulk density, soil porosity, and temperature are amongst the seven physical properties of soil; hence harvesting and logging has an impact on them. The bulk density of the soil is defined as the mass per unit volume of the soil and represents the ratio of the mass of solids to the total or bulk volume of the soil [6]. Soil porosity is that part of the bulk volume not occupied by either mineral or organic matter but it is an open space occupied by air or water while soil temperature is the thermal diffusivity or conductivity of soil at different weather conditions [6].

According to [4], soil physical properties are of great importance. For instance, soil bulk density reflects the soil's ability to function for structural support, water and solute movement, and soil aeration. In addition, soil temperature plays an important role in many processes, which take place in the soil such as chemical reactions and biological interactions. The metabolic activity of soil microorganisms, seed fermentation, and plant roots is directly influenced by soil temperature in terms of water movement and soil freezing [7].

*Pinus kesiya *Royle ex Gordon occurs naturally in Himalaya region (Asian): Burma, China, India, Laos, Philippines, Thailand, Tibet, and Vietnam [8]. This species particularly grows well at altitudes from 600 to 1800 m above sea level [9]. The trees can reach heights of 30–35 or 45 m with straight, cylindrical trunk [10].* Pinus kesiya *is a major exotic plantation species in Malawi and other southern African countries. Its success as an exotic is due to its fast growth rate and wide adaptability [11].

Current studies have shown a large degree of site specificity both in soil and tree growth responses to soil compaction [12, 13]. These research results have encouraged new intuitions on the effects of soil compaction on forest productivity in different soil types and proved the importance of having site-specific soil quality assessments in forests. Moreover, there is still limited information on the relationships between soil compaction/disturbances and tree growth. Generalization about negative effects of harvest-related soil disturbance on tree growth may be in error because these impacts depend on their type and severity and on soil properties and climatic conditions [12, 14]. In addition many studies have assessed tree growth and soil response in logged sites using retrospective approach [15], which may not allow ascertaining the original type, degree, and extent of disturbance [12]. Therefore, the present study was undertaken to determine the impacts of soil disturbance and compaction on soil physical properties and tree growth and the effectiveness of tillage in maintaining or enhancing site productivity for intensively managed* Pinus kesiya* sites in the study area using prospective approach.

## 2. Materials and Methods

### 2.1. Study Site

The study was conducted in Malawi located in Southern Africa in the tropical savannah region at Chongoni plantation (14° 19′ S, 34° 16′ E, and 1650 m above sea level). The climate of the area is humid tropical with distinct dry and rainy seasons. The rain season starts from around mid-November to late April and the rainfall pattern is bimodal with peak periods in January and February. Chongoni plantation receives 1200 mm to 1800 mm rainfall per annum, with annual temperature ranging from 7°C to 25°C. The dry season runs from May to October. It is situated about 85 km southeast of Lilongwe the capital. In addition, the experimental site has homogenous soil and topographic conditions. The site is on gentle slope (<10%); the soils are deep, well-drained, mostly stone-free, and high in ferralsols, acrisols, and nitosols, with site index of 20 m at 25 years for* Pinus kesiya* [16].

### 2.2. Experimental Design

The old-growth stand previously occupying the site was planted in 1980 with* Pinus kesiya* and was harvested from May to August 2010. This is the period when this study was installed. The study contained three treatments replicated four times in a randomised complete block design. Treatments plots are 70 m by 70 m (0.49 ha). The treatments were as follows:

T1: stem-only removal with soil compaction;
T2: stem-only removal with soil compaction plus tillage;
T3: stem-only removal with no soil compaction (control).

The stem-only removal harvest followed merchantability standards of 5.5 m log length and 8 to 13 cm small end top diameter (i.e., cut-to-length (CTL) operation). Logging slash was scattered uniformly across each plot during the log-forwarding operation.

The harvested trees were directionally hand-felled between June and August 2010 so that all tree tops remained within the plot. In T1 and T2 logs were pulled by a tractor from the stump area to the road side before being transported to the sawmill. In T2 further thorough soil tillage to the depth of 60 cm was accomplished by using a plough. In T3 sulkies were used to remove the logs from the stump area to the road side. Sulkies were used in order to minimise site disturbance. Ten equally spaced traffic lanes were identified in each plot to be trafficked to make soil disturbance comparable across blocks. Every other lane was trafficked twice to more closely mimic traffic patterns that operationally occur when a tractor or a sulky is used during harvesting.

In December 2010, the plots were planted with* Pinus kesiya* seedlings on a 2.75 m by 2.75 m spacing (1320 trees ha^−^) using hole planting. The experimental area was fenced to eliminate cattle and goat browsing. All treatments including spot weeding in the three growing seasons after planting were carried out in order to eliminate the confounding effects that soil disturbance and compaction can have on vegetation communities and competition pressure [12].

### 2.3. Data Collection

#### 2.3.1. Soil Bulk Density

Twenty (20) soil samples from each treatment were collected to a depth of 0–10 cm and 10–20 cm in September 2010 using a 31.2 mm diameter punch tube volumetric sampler. According to Walworth [17], soil samples of 15 to 25 collected from randomly selected locations in a field are a good representative of the entire field. Simple random strategy was used to avoid bias in collection of the samples. Samples with significant amounts of sound or decayed wood material were discarded and replaced with new samples. In compacted and compacted and tilled plots, sampling was restricted to traffic lanes. Soil samples collected were then oven-dried at 105°C for determination of bulk density and soil gravimetric and volumetric water content.

#### 2.3.2. Soil Disturbance

Soil disturbance conditions were measured in September 2011. The measurements were taken at the point where each tree was planted and the rut depth from the original soil level was recorded. Furthermore, around every other measurement tree, the percentage of each disturbance class in a tree centred 2.75 by 2.75 m square area was recorded. Then the percentage of the ground area and the tree frequency in each soil disturbance class was calculated.

#### 2.3.3. Soil Strength

Soil strength was measured in September 2012 in twenty locations per plot using a cone penetrometer at 5 cm intervals to the 60 cm depth on each treatment. Cone index values were read using a scale template aid over the penetrometer cards. High outlier values created when roots or buried wood was hit by the penetrometer were noted on the cards in the field and those measurements were discarded from the dataset.

#### 2.3.4. Soil Porosity and Water Retention Curves

Soil porosity and water retention curves were determined in October 2012. Soil cores were randomly collected in all the treatments using 75 cm^3^ cylinder rings centred at 5 cm and 15 cm depths to characterize the 0–10 cm and 10–20 cm depths, respectively. Twenty cores per depth were taken in all plots. Soil water retention curves and particle density were determined at Science Laboratory of Malawi College of Forestry and Wildlife. Total porosity was determined by both the gravimetric method with water saturation [12] and the particle density method [18]. Volumetric water contents were determined after equilibrating the soil with water at tensions of −10, −20, −200, −400, and −1500 kPa. Pore size distribution and water retention were then determined by the procedure as outlined by Ares et al. [12].

#### 2.3.5. Soil Temperature

Measurements of soil temperature were taken monthly between 08.00 hours and 10.00 hours from January 2011 to December 2013 at 20 cm depth in all the treatment plots. The measurements were taken using a digital temperature logger.

#### 2.3.6. Tree Measurements

Trees in all treatments were measured immediately after planting and yearly in the three consecutive growing seasons. The parameters measured were total height (h) using a telescopic pole, stem basal diameter (bd) measured at a permanently marked location 15 cm above the ground level in growing seasons one through three, and stem diameter (dbh) at 1.3 m above ground in the growing seasons two and three. Stem diameters were measured using a diameter tape. A stem volume index (SVI) was calculated as (bd)^2 ^h. Mortality of trees in all the treatments was also assessed at year 3.

### 2.4. Statistical Analysis

Data obtained were subjected to Kolmogorov-Smirnov D and normal probability plot tests using Statistical Analysis of Systems software version 9.1.3 [19]. This was done in order to check the normality of the data. Then, harvesting and logging effects on soil strength and bulk density were analysed as a mixed model of repeated measures data with soil depth and soil disturbance as fixed effects and block as a random effect [20]. The model was fitted using the MIXED procedure in SAS [19], which estimates variance components using restricted maximum likelihood methods. Coefficients of the van Genuchten equation [21] were calculated using NLIN procedure in SAS [19]. Differences between treatment means were separated using Fischer's least significant difference (LSD) at the 0.05 level.

A repeated measures analysis was appropriate because measurements of bulk density and soil strength were done at different depths at the same sampling point, and, therefore, sampling errors were not independent. Measurements taken at adjacent depths are expected to be more correlated than measurements taken some distance apart [12].

The covariance structures associated with the within subject factor were selected by choosing those with lowest value for the Bayesian Criterion and Akaike's Information Criterion. The first order autoregressive heterogeneous covariance structure provided the lowest values for both criteria. The arc-sine square root transformation was used for data in percentage. A mixed model approach was also used to test for soil disturbance effects on tree growth in years 0 to 3.

## 3. Results

### 3.1. Soil Disturbance and Physical Properties

**Figure 1:**
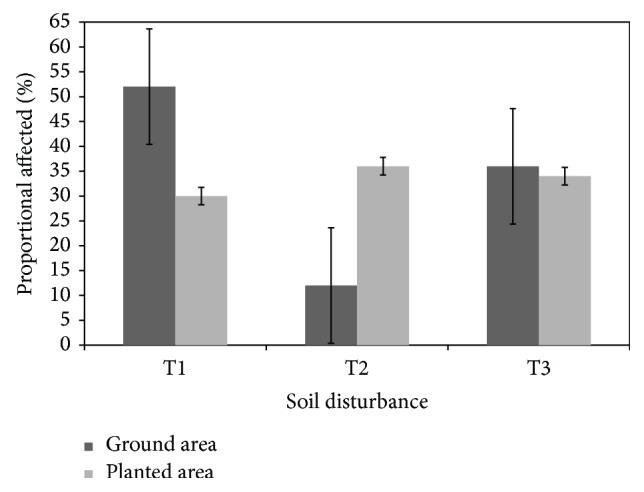
Area in each treatment and proportion of tree seedlings planted on each treatment at the study site.

The result for ground area affected by the soil disturbance is presented in Figure 1. The results indicate that about fifty-two percent of the area of compacted plots was affected by the vehicular traffic. Seventy percent of the trees within the measurement plots of the compacted treatment were located on some degree of soil disturbance. Rut depths were shallow to moderate with averages of 2.8 cm for T2 and 13.1 cm for T3, respectively.

Soil bulk density as related to soil depth and soil disturbance in the study site is presented in Table 1. The results show that there was a significant (*P* < 0.001) difference for soil bulk density between soil depths. The depth of 10–20 cm had higher soil bulk density than the 0–10 cm depth. Similarly, there was a significant difference for soil bulk density among treatments, with T3 having a higher soil bulk density than T1 and T2. Relative to T1, soil bulk density at the depth of 0–10 cm increased 28.9% for T2 and 48.9% for T3. At the depth of 10–20 cm, the increase in soil bulk density was 22.2% and 38.9% for T2 and T3, respectively, in relation to T1. Soil bulk density significantly (*P* < 0.001) increased with both soil disturbance and depth.

**Figure 2:**
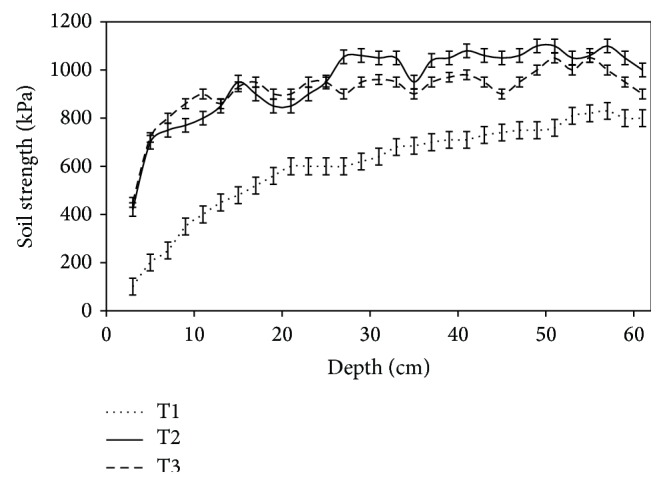
Soil strength by treatment at the study site.

Soil strength by soil disturbance classes is presented in Figure 2. The results indicate that soil strength significantly (*P* < 0.001) increased with soil disturbance and depth, with T1 producing a lower soil strength than class T2 and T3. However, there was no significant (*P* > 0.05) difference for soil strength between T2 and T3 with increase in depth.

Total soil porosity for all the treatments related to soil depth in the study sites is given in Table 2. The results indicate that there was no significant (*P* < 0.05) difference for soil porosity between soil depth, even though the depth of 0–10 cm produced higher soil porosity than 10–20 cm depth. There was a significant (*P* < 0.001) difference for soil porosity among the different treatments with noncompacted soil producing higher soil porosity than the compacted and compacted plus tillage treatments in both soil depths of 0–10 cm and 10–20 cm, respectively. However, there was no significant (*P* < 0.05) difference between noncompacted and compacted plus tillage treatments in soil porosity in both soil depths of 0–10 cm and 10–20 cm, respectively. On compacted soil, total porosity at 0–10 cm depth decreased by 13.8% compared with noncompacted soil and by 16.1% at the 10–20 cm depth.

**Figure 3:**
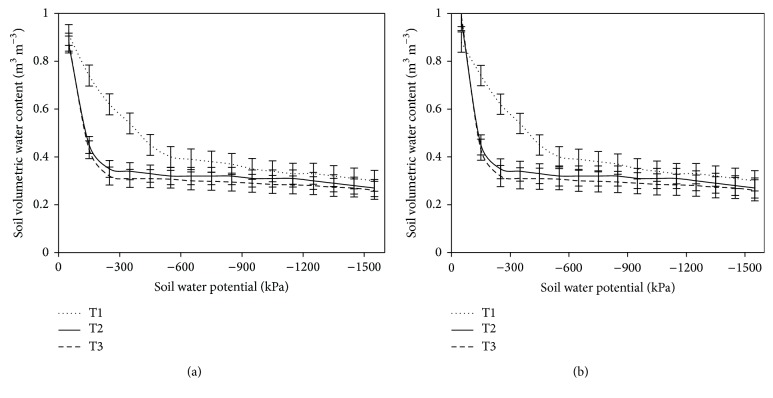
Water retention curves for all the treatments at the (a) 0–10 cm and (b) 10–20 cm depths at the study site. T1: compacted soil; T2: compacted soil plus tillage; T3: noncompacted soil.

Soil water retention curves for all the treatments are presented in Figure 3. The results indicate that there was a significant (*P* < 0.001) difference in water retention by different treatments with compacted soil consistently retaining more water at −20 to 400 kPa water potential than noncompacted or compacted plus tillage soil at both 0–10 cm and 10–20 cm depth.

Soil temperature, measured once a month at 0–20 cm depth, was not significantly (*P* > 0.05) different between compacted and noncompacted areas (data not shown).

### 3.2. *Pinus kesiya* Mortality and Growth

Tree mortality and growth for* Pinus kesiya* throughout the three growing seasons are presented in Table 3. The results indicate that there were no significant (*P* > 0.05) differences in mean height, stem basal diameter, diameter at breast height, stem volume index, and mortality for trees growing on compacted, compacted plus tillage, and noncompacted treatments at years one to three.

## 4. Discussion

### 4.1. Soil Disturbance and Physical Properties

This study illustrates that the amount of soil disturbance in cut-to-length (CTL) operation by a tractor is about forty-eight percent. The results are within the range reported by [22–24]. Tepp [22] reported a 38% soil disturbance using a CTL harvester and forwarder, while Geist and Cochran [23] found that tractor harvesting using CTL produced 36% soil disturbance. Froehlich [24] determined that during tractor logging operations, 50% of soil disturbance occurred. However, the present study results are higher than those reported by [25, 26]. Armlovich [25] reported that the amount of soil disturbance in a cut-to-length (CTL) operation had an average disturbance of 23.2% while in a study by Gingras [26] average disturbance was 25%. This difference may arise because in the present study, equipment traffic was applied when soil water contents were at somewhat drier than field water capacity, which is considered to correspond to a given soil water potential [12, 27]. According to Heninger et al. [28] and McNabb and Boersma [29], the degree of soil disturbance depends on the intensity of site treatment, type of equipment used, time of year when harvesting occurs, frequency of entry, amount of slash or litter on site, soil moisture, and soil type.

Soil bulk density significantly (*P* < 0.001) increased with both soil disturbance and depth. The results are in agreement with those reported by [12, 30]. According to Froese [31], in the upper soil, biological activity (roots, animals, etc.) can act to reduce resistance and soil bulk density while at lower depths soil texture, gravel content, and structure may increase soil resistance and soil bulk density; hence soil bulky density increases with depth. Soil bulk density also increases with an increase in soil compaction [32].

Soil strength in this study also increased after vehicle trafficking to about 60 cm depth but it was not detrimental for tree root growth of 2000 to 3000 kPa depending on tree species and soil conditions [12, 31, 33]. The mean soil strength by depth increment in the study site never exceeded 1200 kPa.

The compacted soils decreased the total soil porosity at the study site by 13.8% and 16.1% in the depths of 0–10 cm and 10–20 cm, respectively, compared to the noncompacted soils. These results are within the range of those reported by [12, 34]. According to Kim [35], the decrease of soil porosity in T2 as compared to T1 is because of soil compaction. In T2 soil is highly compacted due to soil texture and structure by determining the size, number, and interconnection of pores. Coarse-textured soils have many large (macro)pores because of the loose arrangement of larger particles with one another. Fine-textured soils are more tightly arranged and have more small (micro)pores [36]. Macropores in fine-textured soils exist between aggregates. Because fine-textured soils have both macro- and micropores, they generally have a greater total porosity, or sum of all pores, than coarse-textured soils [37].

Water retention increased by soil compaction. This is in agreement with the results reported by [12, 38]. According to Ares et al. [12] compacted soil retained more water than noncompacted soil because compression converted larger pores to smaller ones, especially in the upper part of the soil profile.

### 4.2. *Pinus kesiya* Early Growth

The study reveals that mean height, stem basal diameter, diameter at breast height, stem volume index, and mortality for* Pinus kesiya* trees species were not reduced by soil disturbance or compaction despite the fact that the soil physical properties were affected. Values for these growth indicators were even greater for trees on disturbed soil than those on nondisturbed soil in years 2 and 3. The results are in line with those reported by [12, 39]. Ares et al. [12] reported that Douglas-fir growth was not reduced by soil disturbance and compaction even though the soil physical properties were affected from ground-based harvest.

According to Ares et al. [12], since the growth was not reduced by the soil disturbance or compaction, this is an indication that bulky density, soil strength, and macroporosity did not reach levels in compacted areas that can reduce tree growth. The observed bulk density in the compacted area in this study ranged from 0.45 to 0.66 Mg m^−3^, which is within the 0.50 to 1.11 Mg m^−3^ range for* Pinus kesiya* seedlings growth [40, 41]. Furthermore, the soil strength observed in this study did not exceed 1200 kPa; that is, it remained well below the critical threshold for tree root growth considered to be around 2000 to 3000 kPa depending on tree species and soil conditions [12].

## 5. Conclusion

Soil physical properties particularly soil bulk density, soil strength, and total soil porosity were greatly affected by compaction which was brought by harvesting and logging. However, the extent of disturbance was not detrimental for early growth of planted* Pinus kesiya*. The high organic matter content, low bulk density, and low compressibility of the soil contributed to buffering the harvest impacts and made this soil conducive for intensive forest management and ground based harvesting systems. Soil compaction could also be considered to be beneficial to early tree growth at three years. Increased water available in the −20 to −400 kPa range on compacted traffic lanes may explain this increased growth.

## Figures and Tables

**Table 1:** Bulk density (mg m^−3^) with standard errors in parenthesis as related to soil disturbance and soil depth at the study site.

Soil depth (cm)	Soil disturbance		
	Compacted	Compacted plus tillage	Noncompacted
0–10	0.45 (0.01)^bz^	0.58 (0.01)^by^	0.67 (0.02)^bx^
10–20	0.54 (0.01)^az^	0.66 (0.01)^ay^	0.75 (0.02)^ax^
LSD	0.02		
CV%	3.1		

Note: means with different letter differ (*P* < 0.001). The first letter after each mean refers to comparison between depths and the second letter to comparison among soil disturbances.

**Table 2:** Total soil porosity (m^3^ m^−3^) with standard errors in parenthesis as related to depth at the study site.

Soil depth (cm)	Total soil porosity (m^3^ m^−3^)		
	Compacted	Compacted plus tillage	Noncompacted
0–10	0.50 (0.006)^ay^	0.55 (0.005)^ax^	0.58 (0.005)^ax^
10–20	0.47 (0.005)^ay^	0.53 (0.005)^ax^	0.56 (0.005)^ax^
LSD	0.01		
CV%	3.8		

Note: means with different letter differ (*P* < 0.001). The first letter after each mean refers to comparison between depths and the second letter to comparison among treatments.

**Table 3:** *Pinus kesiya* height, stem diameter, and volume index (SVI) at the end of three growing seasons in different treatments at the study site.

Variable	Year 1			Year 2			Year 3		
	T1	T2	T3	T1	T2	T3	T1	T2	T3
Height, cm	92.1^a^	92.0^a^	92.4^a^	156.8^a^	159.4^a^	153.3^a^	269.5^a^	273.8^a^	265.1^a^
Basal diameter, mm	17.6^a^	17.7^a^	17.6^a^	34.2^a^	35.1^a^	33.5^a^	59.2^a^	59.6^a^	58.8^a^
Diameter at breast height, mm	—	—	—	12.7^a^	12.7^a^	12.8^a^	26.3^a^	26.5^a^	25.9^a^
Stem volume index, cm^3^	312^a^	315^a^	313^a^	2034^a^	2163^a^	1920^a^	9693^a^	9926^a^	9366^a^
Trees with measurable dbh, %	—	—	—	11.5^a^	11.8^a^	10.2^a^	98.9^a^	98.5^a^	97.4^a^
Tree mortality, %	—	—	—	—	—	—	2.3^a^	2.4^a^	2.3^a^

Note: means followed by the same letter are not significantly (*P* > 0.05) different among treatments for a given year and tree variable. T1: compacted; T2: compacted plus tillage; T3: Noncompacted.
